# Shared Decision-Making for Patients with Stroke in Neurocritical Care: A Qualitative Meta-Synthesis

**DOI:** 10.1007/s12028-024-02106-y

**Published:** 2024-08-27

**Authors:** Hui Zhang, Carmel Davies, Diarmuid Stokes, Deirdre O’Donnell

**Affiliations:** 1 Nursing Department, Jining No.1 People’s Hospital, Health Road No.6, Rencheng District, Jining, 272000 China; 2 School of Nursing, Midwifery, and Health Systems, University College Dublin, Dublin, Ireland; 3 Center for Interdisciplinary Research, Education, and Innovation in Health Systems, University College Dublin, Dublin, Ireland

**Keywords:** Stroke, Shared decision-making, Critical care, Qualitative research

## Abstract

Decision-making for patients with stroke in neurocritical care is uniquely challenging because of the gravity and high preference sensitivity of these decisions. Shared decision-making (SDM) is recommended to align decisions with patient values. However, limited evidence exists on the experiences and perceptions of key stakeholders involved in SDM for neurocritical patients with stroke. This review aims to address this gap by providing a comprehensive analysis of the experiences and perspectives of those involved in SDM for neurocritical stroke care to inform best practices in this context. A qualitative meta-synthesis was conducted following the methodological guidelines of the Joanna Briggs Institute (JBI), using the thematic synthesis approach outlined by Thomas and Harden. Database searches covered PubMed, CIHAHL, EMBASE, PsycINFO, and Web of Science from inception to July 2023, supplemented by manual searches. After screening, quality appraisal was performed using the JBI Appraisal Checklist. Data analysis comprised line-by-line coding, development of descriptive themes, and creation of analytical themes using NVivo 12 software. The initial search yielded 7,492 articles, with 94 undergoing full-text screening. Eighteen articles from five countries, published between 2010 and 2023, were included in the meta-synthesis. These studies focused on the SDM process, covering life-sustaining treatments (LSTs), palliative care, and end-of-life care, with LST decisions being most common. Four analytical themes, encompassing ten descriptive themes, emerged: prognostic uncertainty, multifaceted balancing act, tripartite role dynamics and information exchange, and influences of sociocultural context. These themes form the basis for a conceptual model offering deeper insights into the essential elements, relationships, and behaviors that characterize SDM in neurocritical care. This meta-synthesis of 18 primary studies offers a higher-order interpretation and an emerging conceptual understanding of SDM in neurocritical care, with implications for practice and further research. The complex role dynamics among SDM stakeholders require careful consideration, highlighting the need for stroke-specific communication strategies. Expanding the evidence base across diverse sociocultural settings is critical to enhance the understanding of SDM in neurocritical patients with stroke.

*Trial registration* This study is registered with PROSPERO under the registration number CRD42023461608.

## Introduction

A stroke is a sudden neurological deficit or loss of function caused by acute focal injury to the central nervous system, primarily due to cerebrovascular disorders [1]. Globally, stroke remains the third-leading cause of combined death and disability, imposing a substantial burden on individuals, families, and society as a whole [2, 3]. Severe stroke cases often require neurocritical care, wherein comprehensive medical care and specialized neurological support are provided to patients with life-threatening stroke conditions. This is a well-organized subspecialty provided in dedicated units or designated beds within general intensive care units (ICUs) [4].

Decision-making for patients with stroke in the neurocritical phase poses unique challenges. Firstly, there’s considerable uncertainty in forecasting the outcome, ranging from complete recovery to varying degrees of functional impairment [5]. This uncertainty necessitates careful consideration of potential outcomes and their implications. Additionally, patients with stroke in neurocritical care often experience reduced decision-making capacity or challenges in communicating their decision preferences due to impaired consciousness or sedation. Very often, patients and treating health care professions rely on surrogate decision-makers, typically family members, to express decision-making preferences, adding complexity to the process [6, 7]. Moreover, the sudden onset of stroke may leave both the patient and surrogate unprepared for decision-making, leading to heightened stress and emotional burden, further complicating the process [5, 7].

Shared decision-making (SDM) is an increasingly endorsed model for health care decision-making [8]. In critical care, SDM is defined as “a collaborative process that allows patients, or their surrogates, and clinicians to make health care decisions together, taking into account the best scientific evidence available, as well as the patient’s values, goals, and preferences” [9]. According to synthesized guidelines from the World Stroke Organization (WSO), it is recommended that at all levels of stroke services, the management of patients with severe stroke should involve the patient (if possible) and their family in SDM, considering the anticipated prognosis of functional recovery [10]. Guidance from the American Heart Association/American Stroke Association (AHA/ASA) and the Neurocritical Care Society (NCS) also underscores the importance of sharing early, timely, and tailored information with critically ill patients with stroke and their surrogates and incorporating their preferences in decisions [11–13].

Decision-making for patients with stroke during neurocritical care often pertains to the continuation or limitation of life-sustaining treatments (LSTs), which greatly impact mortality rates [5, 14]. Furthermore, individuals’ subjective evaluation of the acceptability of disability versus death varies widely, making these decisions highly preference sensitive and necessitating a careful approach to SDM [6, 7]. In the context of neurocritical care, SDM involves various stakeholders, including patients, surrogate decision-makers, and health and social care professionals (HSCPs), each facing distinct challenges [5–7]. Patients with decision-making capacity may differ in their readiness to receive information and ability to process it amid significant health changes [15, 16]. Family members, often supporting patients’ decision-making capacity or acting as surrogate decision-makers, may endure emotional and physical burdens due to the irreversible consequences of decision outcomes [17, 18]. HSCPs, encompassing a diverse range of professionals such as doctors, nurses, rehabilitators, and social workers, encounter challenges in prognostic communication and conflict resolution, leading to emotional distress when navigating inappropriate decision-making options [19, 20]. Throughout this article, “surrogates” and “families” are used interchangeably to denote those involved in SDM on behalf of the patient.

Understanding the experiences and perspectives of those involved in decision-making in neurocritical stroke care is crucial for elucidating how effectively SDM can facilitate goal-concordant care while alleviating decision-making burdens. However, there is a noticeable gap in systematically synthesizing evidence regarding the experiences and perceptions of key stakeholders in neurocritical care decision-making. This review aims to address this gap by using a qualitative meta-synthesis approach to answer the following question: What are the experiences and perceptions of key stakeholders engaged in SDM for neurocritical patients with stroke? Through a comprehensive exploration of stakeholder experiences, this study seeks to provide some critical insight on the essential elements, relationships, and behaviors influencing the complex phenomenon of SDM in contexts of neurocritical care.

## Methods

### Design

This review followed the methodological guidelines provided by the Joanna Briggs Institute (JBI) for systematic reviews of qualitative evidence [21]. Additionally, the thematic synthesis approach outlined by Thomas and Harden [22] was employed, emphasizing transparent connections between the review’s findings and primary studies. This study was registered with PROSPERO (registration number: CRD42023461608), and comprehensive reporting was ensured by adhering to the updated Preferred Reporting Items for Reviews and Meta-Analyses (PRISMA) checklist [23].

### Eligibility Criteria

This review included individuals with cerebrovascular-origin ischemic or hemorrhagic stroke, primarily including cerebral infarction, intracerebral hemorrhage (ICH), or subarachnoid hemorrhage (SAH). To ensure relevance and comprehensiveness, studies related to neurological disorders that specifically address the stroke population or cover patients with stroke were considered for inclusion. Evidence discussing decision-making scenarios involving HSCPs, patients with stroke, and/or their family or decision-supporters was included to align with the concept of SDM. We included studies exploring the experiences, emotions, viewpoints, and perceived challenges and obstacles encountered by stakeholders during the SDM process. The study context encompassed the neurocritical care phase, typically corresponding to the acute phase of stroke care. Locations varied and included dedicated neurocritical care units or general/medical/surgical ICUs, depending on local practice. This review analyzed qualitative data from various methodologies, including phenomenology, grounded theory, ethnography, and mixed-method studies. Detailed inclusion and exclusion criteria are outlined in Table 1.

**Table 1:** Inclusion and exclusion criteria

PICO	Inclusion criteria	Exclusion criteria
Population	Patients with ischemic or hemorrhagic stroke Studies can focus on either patients with stroke or patients with neurological disorders, including stroke Study subjects may include patients, surrogate decision-makers, and HSCPs	Pediatric patients (< 18 years)
Phenomena of interest	Decision-making involves both HSCPs and service users Information on prognosis, treatment options, and health care preferences was shared and exchanged Studies aim to explore stakeholder experiences, emotions, viewpoints, challenges, and obstacles in decision-making	Studies exclusively examine decision-making by either the service provider or the user, without information interaction between both parties
Context	The study context encompasses hospital-based neurocritical care, involving specialized neurological support for life-threatening stroke conditions May occur in various locations, including dedicated neurocritical care units or general/medical/surgical ICUs, depending on local practices	Studies focused solely on the hyperacute phase (typically within 24 h in the emergency department), postacute phase, or chronic phase
Types of study	Qualitative studies (phenomenology, grounded theory, ethnography, etc.) Mixed-method studies with qualitative data	Quantitative studies, reviews, opinion pieces, commentaries, book chapters, and conference abstracts

HSCPs, health and social care professionals, ICU, intensive care unit

### Search Strategy

A senior librarian at University College Dublin (DS) guided the development of the search strategy. Following the PICo mnemonic [21] (population, phenomena of interest, and context), three search strings were created. Initially, the keywords were searched in PubMed and CINHAL to identify subject terms and more relevant keywords. Subsequently, searches were conducted across five databases, PubMed, CIHAHL, EMBASE, PsycINFO, and Web of Science, using a combination of subject terms and keywords tailored to each database, with a last search date of August 22, 2023. Additionally, a manual search was performed on Google Scholar and the official websites of relevant international organizations, including WSO, AHA/ASA, NCS, and the International Shared Decision-Making Society, to uncover potentially unsearched and gray literature. Furthermore, during the full-text search phase, the reference lists of included studies were reviewed, and a forward citation search was conducted to identify any additional eligible studies.

Considering the language proficiency of the research team, the included studies were limited to those published in English and Chinese, without restrictions on publication dates. The detailed search strategy and record is provided in Appendix A1.

### Selection Process

**Fig. 1:**
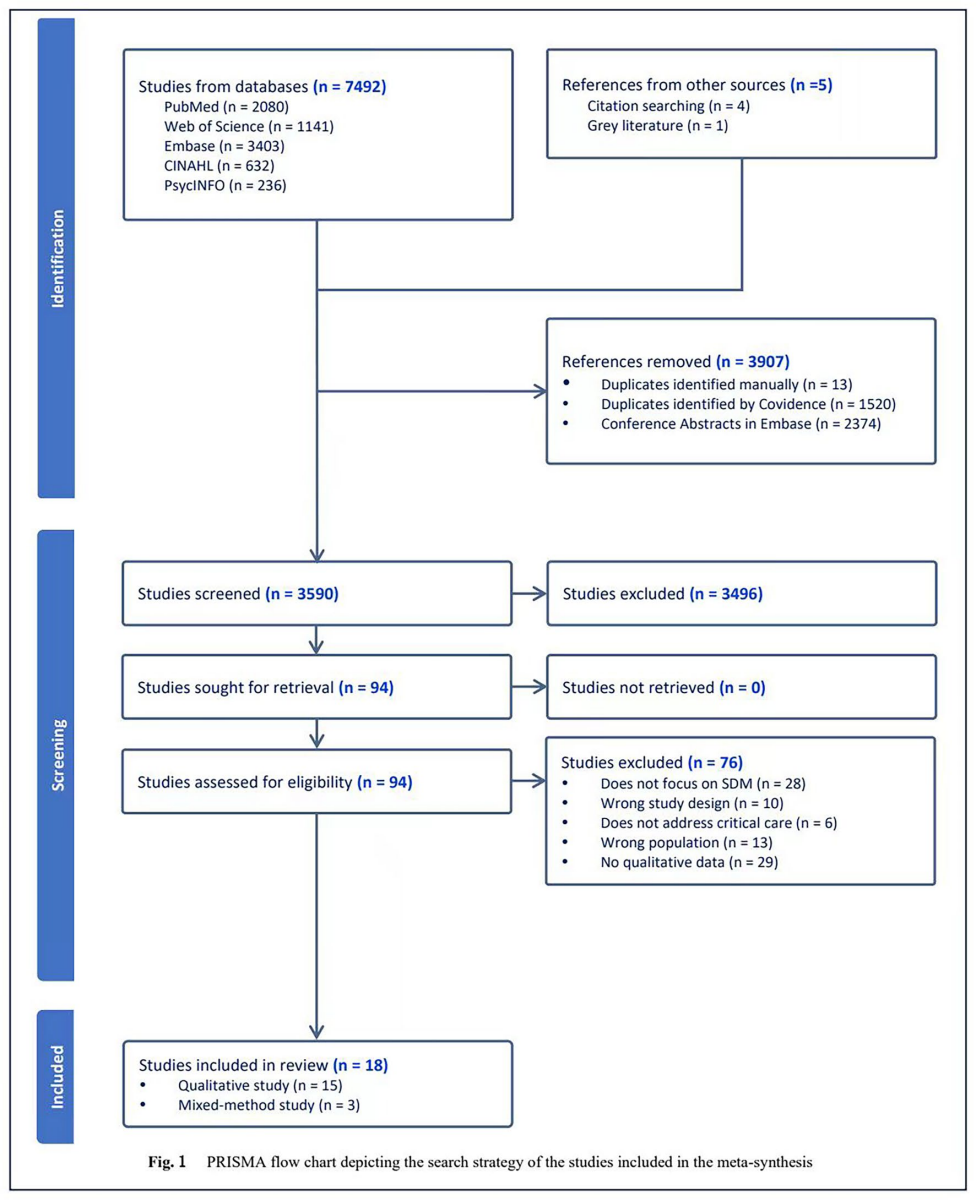
This flowchart represents the process of literature inclusion following the standard PRISMA format. It provides a clear overview of the data sources and the literature screening steps. A total of 94 articles were screened in full text, with 76 being excluded for not meeting the inclusion criteria. This left 18 articles that were ultimately included in the final meta-synthesis. The flowchart also details the specific reasons for excluding articles during the full-text assessment stage. PRISMA Preferred Reporting Items for Systematic reviews and Meta-Analyses, SDM shared decision-making

The Covidence software facilitated the selection process [24]. Initially, a team of three reviewers (HZ, DOD, and CD) conducted a pilot screening of 50 documents to ensure a consistent understanding of inclusion criteria based on a shared definition of the target population, phenomenon of interest, and context (see Table 1). Subsequently, HZ screened titles and abstracts, with any uncertainties proceeding to full-text screening. The full texts of all potentially eligible articles were obtained for further assessment. Independent full-text assessments were conducted by HZ and DOD. Any discrepancies were resolved through discussion or consultation with the third reviewer, CD. Reasons for excluding articles during the full-text evaluation were carefully documented in the PRISMA flowchart (Fig. 1) [23].

### Data Extraction

A preliminary data charting form was developed to extract relevant information related to the research question, encompassing details such as author and publication year, country, aims, study design, data collection methods, stroke types, decision options, and main findings (see Table 2). Prior to formal data extraction, three documents were selected for pilot extraction to ensure accuracy. Based on the pilot results, the charting form was revised to enhance clarity and comprehensiveness. Subsequently, HZ conducted data extraction from the included literature, and DOD verified the accuracy of the extracted information.

**Table 2:** Characteristics of included studies (*n* = 18)

Authors/year, country	Aim	Research design	Data collection	Setting	Participants	Stroke types	Decision options	Main findings
Göcking et al. 2023 [15], Switzerland	To explore the impact of aneurysmal SAH on the informed medical decision-making process from the perspective of the involved parties	Phenomenology	One-by-one semi-structured interviews	A Swiss neurosurgical ICU	Directly affected (*n* = 5), next of kins (*n* = 4), clinicians (*n* = 2)	SAH	General SDM	(1) Making decisions when faced with uncertain prognostic outcomes is most challenging for all groups (2) Although clinicians typically centered their efforts on treatment determination, the service users placed significant value on participating in the SDM process
Visvanathan et al. 2019 [16], UK	To enhance comprehension of treatment decision-making during the initial stages of stroke and to explore the perspectives, encounters, and requirements of stroke survivors both during their hospital stay and six months after the event	A longitudinal qualitative study	One-by-one semi-structured interviews	Stroke unit in a large teaching hospital	Patients (*n* = 15 within a week after admission, *n* = 11 six months later)	Disabling stroke (specific types not reported)	Life-extending treatments	(1) Patients’ levels of functioning prior to their stroke seemed to influence their participation in SDM (2) There is a mismatch between patients’ need to uphold hope for functional recovery and their subsequent desire to have received realistic information in the early poststroke stage, named the “hope-information paradox.”
de Boer et al. 2015 [17], Netherlands	To gather the viewpoint of family members of severe stroke patients during the initial critical stage, aiming to comprehend their involvement in treatment choices	Grounded theory	One-by-one semi-structured interviews	Four hospitals and a nursing home	Relatives (*n* = 15)	Severe stroke (specific types not reported)	Life-and-death decisions	Four distinct categories were identified: (1) deciding amid time constraints, (2) grappling with the question of personal authority in making decisions, (3) hesitancy in consenting to “allowing her to pass away” and (4) managing unforeseen alterations
Goss et al. 2023 [18], United States	To gain a deeper comprehension of surrogate experiences and requirements during the SDM process in order to enhance communication between clinicians and surrogates	Grounded theory	One-by-one semi-structured interviews	ICU at one academic medical center	Surrogate decision-makers (*n* = 22)	SABI (including stroke)	Life-sustaining therapy	(1) There are two distinct perspectives among surrogates: one group exhibited a strong sense of agency around decision-making, whereas the other group expressed a more subdued involvement in decision-making (2) The central challenge identified was the uncertainty surrounding the prognosis (3) Only those surrogates who believed they were actively engaged in decision-making found time-limited trials to be beneficial
Douba et al. 2018 [19], UK	To investigate the primary educational requirements of HSCPs concerning palliative and end-of-life care following stroke	Mixed-method Study	Survey with open and closed questions	Six UK multi-professional networks and two groups of local clinicians	Multi-disciplinary professionals (*n* = 489 in qualitative analysis)	Stroke (specific types not reported)	Palliative and end-of-life care	Five major themes were identified from free-text questions: (1) challenges of prognostic uncertainty, (2) managing stroke-related clinical issues, (3) discussing expectations and priorities, (4) skills for holding conversations, and (5) logistics and infrastructure
Mc Lernon et al. 2020 [20], UK	To undertake a qualitative investigation into how HSCPs perceive neurocritical care concerning the anticipated functional recovery of patients with ICH	Descriptive qualitative study	One-by-one semi-structured interviews	Neurocritical care department in a tertiary referral center	Nurses (*n* = 11), intensivists (*n* = 5), physicians (*n* = 2), neurosurgeons (*n* = 3)	ICH	Life-supporting therapies	Five themes were identified: (1) uncertainty in predicting outcomes, (2) differing interpretations of favorable and unfavorable results, (3) situations in which care is seen as inappropriate, (4) difficulty in making complex decisions, and (5) emotional strain
Visvanathan et al. 2020 [27], UK	To explore the reasons behind the treatment choices made by family members, as well as their information and support requirements	Descriptive qualitative study	One-by-one semi-structured interviews	Stroke unit in a tertiary teaching hospital	Family members (*n* = 24)	Major stroke (specific types not reported)	Life-extending treatments	The ways in which family members make treatment decisions vary along a continuum, influenced by the patient’s health condition and their expressed preferences before experiencing a stroke
Zahuranec et al. 2018 [28], United States	To examine the perspectives of surrogate decision-makers regarding the way healthcare providers communicate prognostic information following ICH	Descriptive qualitative study	One-by-one semi-structured interviews	Hospital wards in five sites	Surrogates (*n* = 52)	ICH	Life-saving measures	(1) Experiencing conflicting prognoses results in emotional distress or feelings of frustration (2) Surrogates found themselves confused by the inconsistent terminology used by health care providers when discussing the diagnosis (3) Surrogates’ responses to uncertainty were diverse
Lank et al. 2023 [29], United States	To identify barriers that Mexican American and non-Hispanic white surrogate decision-makers encounter when applying patient values for life-sustaining treatments	Descriptive qualitative study	One-by-one semi-structured interviews	Community hospitals	Family surrogate decision-makers (*n* = 42)	Ischemic stroke and ICH	Life-sustaining treatments	(1) A small number of surrogate decision-makers had not previously talked about the patient’s wishes (2) Surrogates faced difficulties when attempting to relate previously known values and preferences to specific decisions (3) Surrogates experienced feelings of guilt or burden frequently, even when they had some awareness of the patient’s values or preferences (4) Preserving patient self-reliance stood out as the highest priority
Rejnö et al. 2012 [30], Sweden	To narrate how members of HSCPs in stroke units encounter ethical dilemmas and the strategies they employ when handling stroke patients experiencing sudden and unforeseen fatalities	Descriptive qualitative study	Focus group interviews	Stroke units in four county hospitals	Physicians (*n* = 4), nurses (*n* = 9), enrolled nurses (*n* = 6)	Stroke (specific types not reported)	End-of-life decision	(1) Three themes are give information, decide on care, and provide support for next-of-kin in changing and uncertain situations (2) The “red thread” linking these three themes was mutual trust within the stroke team and with next-of-kin, which was seen as a way of dealing with ethical issues
Tolsa et al. 2022 [31], Switzerland	To explore clinicians' perspectives on applying predictive scores to determine prognosis in severe acute stroke and their perceptions of their own cognitive tendencies	Descriptive qualitative study	Focus group discussions	Stroke units in two hospitals	Physicians (*n* = 21)	Severe stroke (specific type not reported)	Life-sustaining treatments	(1) Although most physicians recognize some value in predictive tools, they are not routinely used in clinical practice after severe stroke; instead, physicians attach more to their clinical experience (2) Physicians reflected that it is common for emotions and cognitive biases to influence decision-making
Seeber et al. 2015 [32], Netherlands	To understand the ways in which neurologists engage family members in the care process and decision-making for patients who lack the capacity to make decisions	Descriptive qualitative study	One-by-one semi-structured interviews	Eight hospitals from different areas	Neurologists (*n* = 20)	Neurological diseases (including stroke)	Treatment restriction decisions	Families are tasked with three distinct yet interconnected roles: (1) providing information about the preferences of patients, (2) actively participating in the provision of care, and (3) experiencing their own emotional distress and challenges. Each family member’s distinct role held significance in end-of-life care and decision-making, as opposed to solely being a legal surrogate
Frey et al. 2020 [33], Netherlands	To conduct an ethnographic investigation into the decision-making processes that occur in the immediate aftermath of a severe stroke	Ethnographic study	Participant observation and interviews	Three stroke units	Cases of severe stroke (*n* = 16)	Stroke (specific type not reported)	Tube feeding	Three different repertoires were discerned: choice, necessity, and comfort, each suggesting unique ethical obligations, primary considerations, information sources, and timelines
Lou et al. 2022 [34], United States	To explore the long-term reflections of patients and their families as they retrospectively consider the choice of undergoing tracheostomy following SABI	Descriptive qualitative study	Semi-structured interviews	30-bed neuro-ICU	Patients (*n* = 1) and family members (*n* = 20)	SABI (including stroke)	Tracheostomy	Two themes emerged: (1) families did not perceive tracheostomy as a decision due to the presence of prognosis uncertainty, (2) families recognized a core requirement for receiving consistent and compassionate communication focused on providing clarity and instilling hope
Kendall et al. 2018 [35], UK	To gain insight into the experiences, concerns, and priorities of patients, as well as informal and professional caregivers, during the year following a total anterior circulation syndrome stroke	Mixed-methods study	One-by-one semi-structured interviews	Three stroke units in central Scotland	Patients, informal caregivers, and HSCPs (99 interviews in total)	Ischemic stroke and ICH	Palliative care	(1) HSCPs concentrated on physical rehabilitation, recovery, motivation, and hope rather than preparing for end-of-life situations or addressing limited recovery prospects (2) The uncertainty surrounding prognosis made it difficult to engage in future planning (3) The term “palliative care” was associated with withdrawing treatment and the perception of imminent death and should be reframed
Kiker et al. 2021 [36], United States	To enhance comprehension of the disagreement in prognosis between physicians and families by assessing its prevalence and identifying the factors linked to it	Mixed-methods cross-sectional study	Survey with open and closed questions	Medical and cardiac ICUs of a single neuroscience center	Family members (*n* = 94 in qualitative analysis)	SABI (including stroke)	General SDM	(1) Divergence in prognosis between physicians and families was frequently observed in patients with SABI (2) Two key themes, named faith and uncertainty, emerged as fundamental factors contributing to the differences in beliefs
Payne et al. 2010 [37], UK	To discern the encounters of individuals affected by acute stroke, as well as the choices they make regarding end-of-life care	Cross-sectional qualitative exploratory study	One-by-one semi-structured interviews	Two district general hospitals with specialist palliative care teams	Patients with stroke (*n* = 28), family members (n = 25)	Ischemic stroke and ICH	End-of-life decision	The two primary themes were (1) communication and the provision of information and (2) confronting uncertainty and addressing end-of-life preferences The manner in which information was conveyed held equal importance as the actual content of the information
Tran et al. 2016 [38], United States	To investigate the delivery of specialized palliative care to individuals experiencing SABI	Qualitative study	Qualitative analysis of electronic health records	A neuro-ICU of a regional stroke and trauma center	Cases of severe stroke (*n* = 25)	SABI (including stroke)	Life-sustaining treatments	The primary themes were (1) prognosis discussion, (2) gathering patient and family values, (3) comprehending medical choices, and (4) recognizing conflicts

HSCPs, health and social care professionals, ICH, intracranial hemorrhage, ICU, intensive care unit, SABI, severe acute brain injury, SAH, subarachnoid hemorrhage, SDM, shared decision-making

### Quality Appraisal

The JBI Appraisal Checklist for Qualitative Research [25] was employed, comprising ten items that assess methodology, research objectives, data collection, data analysis, findings, researcher’s cultural or theoretical positioning, researcher’s influence, participant representation, ethical considerations, and conclusions. Each item was evaluated, and responses were categorized as “yes,” “no,” “unclear,” or “not applicable.” HZ performed the critical appraisal, and DOD conducted a thorough cross-verification of the assessment results. In cases of discrepancies, CD facilitated discussions and led to a consensus on the assessment outcomes.

### Data Synthesis

Thomas and Harden’s thematic synthesis approach involves three steps: initial line-by-line coding, development of descriptive themes, and creation of analytical themes [22]. NVivo 12 software supported the analysis process [26]. Initially, HZ meticulously read and reread each article to gain a comprehensive understanding of the data. The results section of each article was coded line by line. These initial codes were then grouped to form descriptive themes involving the examination of commonalities and disparities among the codes. These descriptive themes were refined through discussions (HZ, DOD, and CD). Subsequently, the descriptive themes were synthesized into analytical themes, aligning with our research goal of exploring the experiences, perceived challenges, and interrelationships of all stakeholders involved in the SDM process. Identification of analytical themes emerged through iterative dialogues among the three researchers, in which theoretical and logical connections between themes were discussed and clarified.

## Results

### Search Results

The initial search yielded 7,492 articles. After deduplication, 3,590 articles underwent title and abstract screening. Of these, 94 articles underwent full-text screening. Seventy-six articles were excluded for not meeting inclusion criteria, leaving 18 articles for the final meta-synthesis. Refer to Fig. 1 for the search and screening process.

### Characteristics of Studies

The included articles originate from five countries, with publication dates spanning 2010 to 2023. Sample sizes varied between 11 and 499 participants. These studies involved patients [16], surrogate decision-makers (referred to as families, family members, relatives, surrogates, or next-of-kin) [17, 18, 27–29], and diverse HSCPs, including physicians, intensivists, neurosurgeons, neurologists, stroke consultants, nurses, enrolled nurses, palliative care specialists, physiotherapists, speech and language therapists, and social workers [19, 20, 30–32]. Seven studies involved multiple decision-making participants [15, 33–38].

Primary decision types included LST (various terms were used, such as “life-prolonging,” “life-supporting,” “life-extending,” or “life-saving”), palliative care, and end-of-life care, with LST being the most prevalent. Treatment options mentioned encompassed admission to the neuro-ICU, hemicraniectomy, resuscitation, tracheal intubation, mechanical ventilation, enteral tube feeding, parenteral fluids, antibiotics, and intermittent pneumatic compression. Two studies specifically focused on tracheotomy and tube feeding [33, 34]. See Table 2 for detailed study information.

### Quality Appraisal Results

Overall, the studies provided adequate descriptive data for an evaluation of rigor. Five studies met all criteria [15, 16, 18, 27, 33]. All research adhered to ethical requirements with formal ethical approval or exemption. The research methodology and data collection methods aligned with the stated research questions and objectives. Data analysis was well delineated, often using an iterative approach combining deduction and induction to enhance credibility. Extensive quoting of participants’ statements ensured effective representation of their voices, grounding conclusions in data.

However, a common weakness was the absence of clear philosophical perspectives. Researchers often conducted qualitative studies based solely on interpretive perspectives without explaining their philosophical assumptions, making it challenging to align philosophical outlooks with methodological choices. Explicit cultural or theoretical orientations were also often missing. Moreover, half of the studies did not critically examine the researchers’ roles and potential impacts during data collection and analysis [19, 20, 28, 31, 32, 34–37]. Despite these shortcomings, all studies were considered eligible for inclusion in the meta-synthesis. An overview of the quality appraisal is presented in Table 3.

**Table 3:** Summary of quality appraisal

References	Q1	Q2	Q3	Q4	Q5	Q6	Q7	Q8	Q9	Q10
Göcking et al. [15]	Y	Y	Y	Y	Y	Y	Y	Y	Y	Y
Visvanathan et al. [16]	Y	Y	Y	Y	Y	Y	Y	Y	Y	Y
de Boer et al. [17]	Y	Y	Y	U	U	U	Y	U	Y	Y
Goss et al. [18]	Y	Y	Y	Y	Y	Y	Y	Y	Y	Y
Douba et al. [19]	U	U	Y	Y	Y	Y	N	Y	Y	Y
Mc Lernon et al. [20]	U	Y	Y	Y	U	Y	N	Y	Y	Y
Visvanathan et al. [27]	Y	Y	Y	Y	Y	Y	Y	Y	Y	Y
Zahuranec et al. [28]	U	Y	Y	U	U	U	N	Y	Y	Y
Lank et al. [29]	U	Y	U	Y	Y	Y	Y	Y	Y	Y
Rejnö et al. [30]	U	Y	Y	Y	Y	U	Y	Y	Y	Y
Tolsa et al. [31]	U	Y	Y	Y	Y	U	U	Y	Y	Y
Seeber et al. [32]	U	Y	Y	Y	Y	Y	N	Y	Y	Y
Frey et al. [33]	Y	Y	Y	Y	Y	Y	Y	Y	Y	Y
Lou et al. [34]	U	Y	Y	Y	Y	U	N	Y	Y	Y
Kendall et al. [35]	U	Y	Y	Y	Y	U	U	Y	Y	Y
Kiker et al. [36]	U	Y	Y	Y	Y	U	N	U	Y	Y
Payne et al. [37]	U	Y	Y	Y	Y	U	U	Y	Y	Y
Tran et al. [38]	U	Y	Y	Y	Y	U	Y	Y	Y	Y

N, no, U, unclear, Y, yes

Q1: Is there congruity between the stated philosophical perspective and the research methodology?

Q2: Is there congruity between the research methodology and the research question or objectives?

Q3: Is there congruity between the research methodology and the methods used to collect data?

Q4: Is there congruity between the research methodology and the representation and analysis of data?

Q5: Is there congruity between the research methodology and the interpretation of results?

Q6: Is there a statement locating the researcher culturally or theoretically?

Q7: Is the influence of the researcher on the research and vice versa addressed?

Q8: Are participants and their voices adequately represented?

Q9: Is the research ethical according to current criteria or for recent studies, and is there evidence of ethical approval by an appropriate body?

Q10: Do the conclusions drawn in the research report flow from the analysis or interpretation of the data?

### Findings

**Fig. 2:**
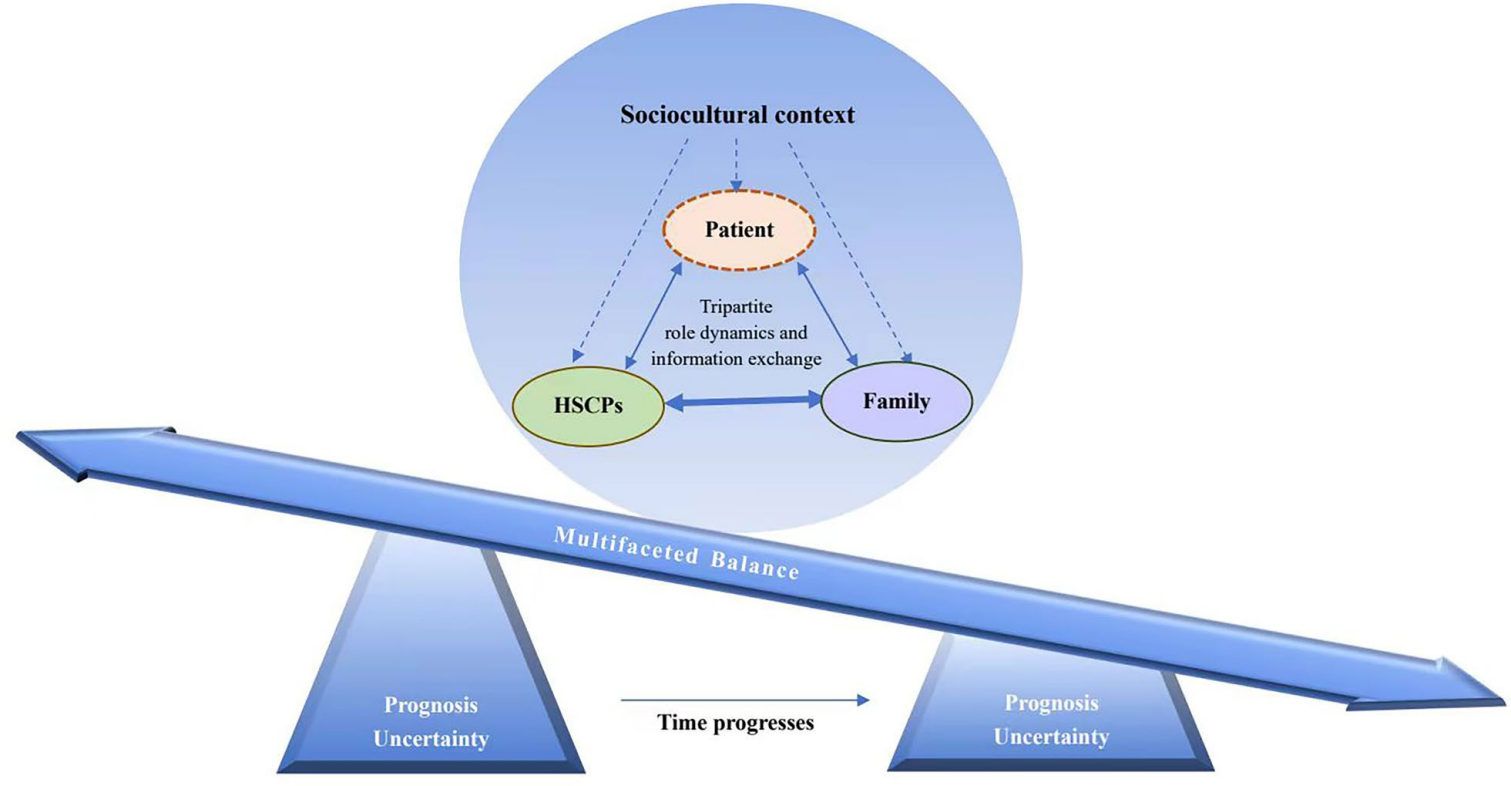
This conceptual model diagram illustrates the complex dynamics of SDM in neurocritical stroke care. At its base is prognostic uncertainty, which acts as the fulcrum around which other SDM elements revolve. Above this foundation, the three key parties involved in SDM (patient, family, and HSCPs) operate within a broader sociocultural context. Prognostic uncertainty serves as the fulcrum for the balance board, and the difficulty of finding equilibrium depends on the level of uncertainty. As time progresses and prognostic uncertainty decreases, the complexity of this balancing process tends to ease. The model also underscores the complex role dynamics and information exchange among the three SDM parties. The thicker line in the diagram indicates that family members and HSCPs typically have more frequent direct interactions, whereas patients may engage less directly. However, the preferences of patients, whether explicitly stated or inferred, remain central to the decision-making process. These interactions are significantly influenced by the sociocultural context, which impacts the experiences of decision-makers, ultimately affecting the outcomes of SDM. HSCPs health and social care professionals, SDM shared decision-making

The synthesis revealed four analytical themes encompassing ten descriptive themes, as outlined in Table 4. Each of these themes is described in detail in this article. Furthermore, a conceptual model (Fig. 2) was developed to visually represent the interrelationships among these themes, providing an abstract depiction of the complex phenomenon of SDM in this context.

**Table 4:** Analytical and descriptive themes identified from the included articles

Analytical themes	Descriptive themes	References
Prognostic uncertainty	Prognostic uncertainty emerges as the primary challenge	[15, 17–20, 27, 28, 30, 31, 34–38]
	Time is crucial for resolving uncertainty	[15–20, 27, 30, 33, 34, 37, 38]
Multifaceted balancing act	Balance between maintaining hope and realism	[15, 16, 18–20, 28, 34–37]
	Balance between participation and responsibility	[15, 17, 29, 30, 32, 33, 35, 37]
	Balance between overtreatment and premature withdrawal	[15, 18, 20, 29–31, 37]
Tripartite role dynamics and information exchange	Patients are often invisible but central	[15, 16, 18, 27–30, 32, 33, 38]
	Family members assume multiple roles	[15, 30, 32, 38]
	Effective information delivery is essential	[15, 16, 18, 19, 28, 30, 32, 34, 35, 37, 38]
Sociocultural context	Social and relational factors influence decision-making dynamics	[15, 16, 19, 27, 28, 32, 33, 37, 38]
	Cultural and religious factors impact preferences and decisions	[15, 18, 29, 36]

### Prognostic Uncertainty: Navigating the Unknown

#### Prognostic Uncertainty Emerges as the Primary Challenge

Thirteen of the 18 articles placed special emphasis on prognostic uncertainty, which was described as the “central challenge,” “most frequently reported concern,” or “a red thread through all the themes” [15, 17–20, 28, 30, 31, 34–38]. This uncertainty has a fundamental influence on the experience of SDM in neurocritical care, profoundly impacting the decision-making process and behaviors of all involved parties.

HSCPs often hesitate to offer prognostic outcomes because of the complex nature of stroke and concerns about these potential outcomes having an undue influence on decisions [19, 20]. Conversely, families seek prognostic estimates to aid in decision-making and future planning [15, 18]. This disparity in information needs leads to frustration and distress for HSCPs and heightened fear, anxiety, and helplessness for surrogates, despite their acknowledgment of the inherent medical uncertainty [15, 18–20, 28, 35, 37]. Given the unpredictability of outcome, some individuals find decision-making to be exceptionally challenging, whereas others feel compelled to proceed with a “just do it” mentality [18, 27, 34]. One study even concluded that “prognostic uncertainty almost transcends the notion of choice” [18].

It’s still really hard to predict what happens from here, and I usually try and say that, you know, some people deteriorate very quickly, some people deteriorate very slowly, some people stabilize and don’t deteriorate particularly. (HSCPs) [35]

It made me anxious. I guess that is probably the best way to describe it. I wanted answers and they really were not able to give me answers. (Families) [28]

#### Time is Crucial for Resolving Uncertainty

Because of the sudden onset of disease characteristics of stroke, patients and families often feel “shocked, overwhelmed, and emotionally unprepared” [15–17, 19, 27, 30]. Consequently, early decision-making is described as “mechanical, passive, and intuitive,” lacking thorough rational considerations [16, 17, 27]. In some cases, discussions about LST decisions occur in advance, particularly for the older population with multiple comorbidities, anticipating potential deterioration in health status. This proactive approach facilitates making early and clear decisions for all SDM participants [27, 33].

However, in most instances, both health care providers and users stress the importance of time, advocating for a cautious approach and advising against rushing into decisions [15, 17, 37, 38]. They recognize that hasty decisions may result in regrettable outcomes. Instead, all involved parties prefer to allow time to serve a supportive role in the decision-making process. They adopt a “wait and see” approach until the minimum acceptable level of recovery becomes evident and the patient’s prognosis is clearer [15, 18, 20, 27, 30, 33, 34]. This approach allows for reassessment and the formulation of new decisions as necessary.

It’s a difficult decision to make, to answer, you need time to think about it, weigh the situation up, and discuss with family members. (HSCPs) [37]

We will give some food and then we will have to wait three days and see how it goes.… And then we can always still say: we will continue feeding, or we stop it. That is also possible. (Families) [33]

### Multifaceted Balancing Act: Negotiating Complex Trade-Offs

#### Balance Between Maintaining Hope and Realism

Hope serves as a crucial coping mechanism, providing faith during desperate times while realistic information helps set reasonable expectations. Families often desired encouraging messages to maintain optimism but were distressed by a lack of honest and forward-looking information, which left them unprepared and led to regrettable decisions [15, 34–37]. HSCPs grappled with balancing hope and avoiding false hope [19, 20]. In the patient-only study, patients retrospectively wished for realistic information in the early phase; however, they initially wanted positive information favoring functional recovery, even if it was inaccurate, a phenomenon termed the “hope-information paradox” [16]. More importantly, patients and families seek uplifting words to cope with stressful circumstances and resent overly negative messages [16, 18, 28, 34].

I want to know I’m going to get back to a hundred percent;… I think it’s vital to move forward…even if it’s not completely true. (Patients) [16]

I got one doctor that just kept saying “never, none, zero” and that was just upsetting. I just personally don’t feel that those words should ever be used in a medical area. (Families) [28]

#### Balance Between Participation and Responsibility

Patient and family involvement in SDM varies, with some feeling excluded and others avoiding it because of the high burden. Patients’ and families’ willingness and self-efficacy to participate in SDM differ widely, requiring HSCPs to adapt their roles as facilitators, collaborators, or directors as needed [15, 17, 37]. In neurocritical care, in which decisions often concern life and death, making decisions may be seen as “playing the role of God” and disrupting the course of nature [33]. Families may hesitate to assume the role of decision-maker, preferring to defer to the “expert knowledge” of physicians [17, 29, 33, 35]. This reluctance may stem from a fear of decision-making accountability and responsibility [17, 29]. Given insights from families’ experiences and dilemmas, HSCPs grapple with balancing participation and responsibility. They often strive to ensure families feel engaged in decision-making without bearing sole accountability, balancing this with the risk of being perceived as paternalistic [30, 32].

You are like now making a decision for somebody else. We do not resuscitate you, that means nobody’s gonna try to help you…they already told me there’s no recovery, but it’s still a hard decision to make, say do not bring this person back.… It’s like I’m trying to be God…I don’t want that role. (Families) [18]

When the situation threatens that the family is forced to decide, you should always try to avoid that, people should not get the feeling that they have to decide about the death of their father or husband. We should take those feelings of guilt away, one way or another. Yes, it is our decision; that’s always the trick. (HSCPs) [32]

#### Balance Between Overtreatment and Premature Withdrawal

Decision-makers face a challenge to balance the intensity of intervention, avoiding both overtreatment and premature withdrawal. Families may express concerns about treatments causing excessive suffering or leaving the patient in an unacceptable state alongside fears of choosing less aggressive measures that may hinder potential recovery [18, 29, 37]. HSCPs share these concerns. They are cautious because of growing awareness of prognostic uncertainty, acknowledging that some patients may recover more extensively than initially predicted. Moreover, the “disability paradox” highlights that certain survivors, despite significant disabilities, express satisfaction with their quality of life. However, HSCPs may also experience moral distress when they perceive patients receiving intervention that they consider inappropriate or unnecessary [15, 20, 30, 31].

The worst part is worrying about her and trying to make decisions about what she would want and the likelihood of her getting back to a life that would be acceptable to her. (Families) [18]

Perhaps the person just has not been allowed to die from an end-of-life event. We are intervening inappropriately to prolong a dying process. (HSCPs) [20]

### Tripartite Role Dynamics and Information Exchange

#### Patients are Often Invisible but Central

Typically, there’s more interaction between family members and HSCPs, with the patient having less direct involvement. Nevertheless, the patient’s preferences and interests remain central to decision-making. Patients with some cognitive capacity may participate directly, whereas those who have diminished decision-making capacity can assert their preferences through preestablished directives, though this is rarely practiced [15, 16, 18, 29, 32, 33, 38].

Frequently, families advocate for patient preferences and make substitute judgments based on their recollections and narratives of the patient’s life stories [18, 27, 29, 30, 32, 33, 38]. Occasionally, patient responses to external stimuli, such as “opening mouth,” “moving hand,” or “pulling out a tube,” are observed to indicate patient preferences and support their involvement in decision-making [15, 27, 28, 33]. Conflicts may be inevitable in some decision-making interactions, arising within health care teams, among family members, and between families and health care teams. In such cases, the patient’s interests should take precedence in conflict resolution [30, 32, 38].

I think [patient] was telling us that by removing the feeding tube and…telling us again by removing the oxygen. (Families) [27]

My task isn’t to please the doctor. I’m speaking for the patient’s best and then there might be conflicts for that reason between me and the doctor because I have a different view…I certainly can fight. (HSCPs, nurse) [30]

#### Family Members Assume Multiple Roles

Because of the impaired or diminished decision-making capacity of neurocritical patients with stroke, family members assume various roles in the SDM process. They act as “supporters” or “surrogates,” playing an active role in SDM on behalf of the patient. In this capacity, they serve as “informants,” conveying patient preferences, advocating for their best interests, and often engaging with HSCPs as “negotiators” for treatment decisions [15, 32].

Additionally, as families navigate changes and potentially face the loss of a loved one, they are also regarded as “sufferers.” Consequently, they become recipients of care themselves [15, 30, 32]. Furthermore, given their potential role as providers of the patient’s future care, family members may be deeply impacted by the decisions made during the SDM process. Therefore, their involvement may be influenced by their own perspectives and interests, further complicating decision-making [15, 30, 32, 38].

Often the next-of-kin would say, “My mum never wanted to become a vegetable, she has said so explicitly,” and some of them say, “Well, mum is so active she is going to live forever.” (HSCPs) [30]

They will have to recognize themselves [in what is decided]. They are the ones who have to live with the decision to fight [for the patients’ life] or not. (HSCPs) [32]

#### Effective Information Delivery is Essential

Patients and families expect to receive useful but not overwhelming information, particularly during the acute phase of illness [15, 16, 32, 37]. They find value in receiving information that includes probabilities and scenario descriptions, such as statements like “never get out of bed” or “20%…she will come back” [15]. It is crucial to tailor information to a level that is easily comprehensible; terms such as “pneumology,” “hospice,” and even “stroke” can unintentionally confuse service users [15, 28, 32, 34].

HSCPs report strategies that promote effective communication, such as repeating key statements and conducting conversations in quiet, private spaces [15, 19, 30]. Additionally, computed tomography scans are a simple yet effective method to help patients and families understand the severity of a stroke [19, 32]. Empathetic and compassionate communication is highly valued by patients and families. They recognize and appreciate supportive communication characterized by kindness and patience, emphasizing the importance of HSCPs not merely treating decision-making as a routine task [34, 35, 37]. HSCPs have faced criticism for their condescending and impersonal communication styles, such as referring to patients as numbers [18, 34, 37]. Importantly, all parties stress the need for clear, consistent, and unified information, as anything less can exacerbate the difficulty of an already challenging decision or even directly influence the choice made [19, 28, 30, 34, 35, 38].

So, it is confusing when you are seeing five different people and they are all telling you five different things. (Families) [28]

I can’t stand doctors that talk down to you. They need to come to your level and explain things if you don’t understand them. And not talk over you, not talk around you, like you’re not in the room. (Families) [34]

### Sociocultural Context: Shaping Perspectives and Choices

#### Social and Relational Factors Influence Decision-Making Dynamics

The patient’s care support system is one of the important considerations in decision-making for HSCPs, with strong support often influencing treatment decisions [32, 33]. HSCPs may gather patient information from various sources beyond the hospital setting, such as general practitioners or home care nurses [32]. Additionally, HSCPs value multidisciplinary discussions with colleagues, finding them beneficial in aiding decision-making processes [15, 38].

Frequently, surrogate decision-makers seek advice and support from a broader network of relationships, including family members, relatives, and friends [27, 28, 37, 38]. Observing and comparing the recovery of peer groups can impact patient and family choices regarding treatment, even though such references may not always align with the perspective of HSCPs [15, 16, 19].

If there is someone who knows the patient well and loves him and says, “Well, if he has a chance of 10 percent [of being able to manage a wheelchair and eat without help] then we should go for it,” yes, that’s decisive. (HSCPs) [32]

In a small community, many of them know other stroke survivors who have made very good recoveries - they assume all strokes are the same and expect their family member to also recover. (HSCPs) [19]

#### Cultural and Religious Factors Impact Preferences and Decisions

Cultural diversity significantly influences treatment preferences among decision-makers from different racial groups, with varying priorities such as valuing individual independence or familial orientation [29]. Individuals from racial minority groups may encounter miscommunication and struggle to establish trust with HSCPs, impacting the SDM process [36].

Religious, spiritual, and faith-based factors also play a significant role in SDM. Patients and family members can find comfort and strength in their faith, aiding them in navigating the challenging decision-making process [15, 18, 36]. However, religious beliefs can sometimes lead individuals to choose certain measures over medical advice, posing challenges for HSCPs involved in the SDM process [15].

And I guess the underlying part of that is that we’re all Christians, and we know what our future is going to hold. (Families) [18]

In this case [case description of a Hindu patient], it was said that he must not die on Fridays because that is somehow not good in Hinduism. (HSCPs) [15]

#### Relationships Between Themes

The conceptual model in Fig. 2 illustrates the relationships between the analytical themes. In the realm of neurocritical patients with stroke, the SDM process unfolds as a multifaceted balancing act deeply rooted in prognostic uncertainty. This involves navigating complex role dynamics and information exchange among the tripartite decision-making body, heavily influenced by the broader sociocultural environment.

Prognostic uncertainty emerges as the primary challenge shaping the trajectory of the SDM process. In the conceptual model, it acts as the fulcrum around which other SDM elements revolve. Given the unpredictable nature of prognosis, time becomes critical in resolving uncertainty and reaching conclusive decisions. Decision-makers navigate challenging trade-offs on a balance board, with prognostic uncertainty as the central pivot. As time progresses and prognostic uncertainty decreases, the complexity of this balancing process may ease.

The SDM process features intricate role dynamics and information exchange among the tripartite decision-makers. Family members and HSCPs interact more directly, depicted by the thicker black line in Fig. 2, whereas patients may have fewer direct interactions but remain central to the decision-making process through their expressed or inferred preferences. Because of the impaired or lost decision-making capacity of neurocritical patients with stroke, family members are actively involved in the SDM process, often assuming multiple roles. The way information is exchanged holds significant importance, frequently having a greater impact on decision-makers’ experiences than the content of the information itself.

These dynamic interactions occur within a broader sociocultural context, comprising factors such as social, relational, cultural, and religious influences. These factors have direct or indirect effects on the experiences of decision-makers and ultimately shape the final outcomes of the SDM process.

## Discussion

This qualitative meta-synthesis is the first to present a conceptual model that illuminates the key elements, relationships, and behaviors influencing SDM in neurocritical care for patients with stroke. The model is based on a systematic synthesis of 18 studies focused on the experiences and perspectives of patients, families, and HSCPs involved in SDM for patients with stroke in neurocritical care. Using a thematic synthesis approach, the study identified four intersecting analytical themes that encapsulate the essence of SDM in this context. These findings align with prior qualitative meta-syntheses on surrogate decision-makers and palliative/end-of-life care in stroke, in which prognosis uncertainty and cohesive communication are similar themes [39, 40]. However, the present study provides additional insights into participants’ role dynamics, multifaceted balancing processes, and the influence of contextual factors.

In this study, prognostic uncertainty was recognized as the key driver shaping the experience of SDM. For neurocritically ill patients, including those with stroke, prognostic uncertainty is well documented, with outcomes ranging from potential full recovery to mortality [39, 41]. Uncertain prognoses complicate the decision-making process significantly and can lead some decision-makers to believe that discussing options is impractical [18, 27, 34]. This hinders efforts to improve the SDM process. Thus, allowing time to play a crucial role is essential for decision-makers to adapt, accept, and make more deliberated decisions based on a clearer prognosis. All parties in the included studies endorsed time as a valuable buffer and support mechanism for SDM [15, 17, 32, 34]. This aligns with professional recommendations for a time-limited observation period to improve prognosis accuracy, given that most stroke-related deaths occur after withholding or withdrawing LST [11, 13]. Consequently, SDM in neurocritical care is not solely about reaching an immediate decision but rather is a process that supports families and patients in adapting, reflecting, and grieving [18, 34]. Over time, repeated conversations can occur based on the patient’s evolving condition and the changing perspectives of the decision-makers.

Effective information exchange is central to SDM [37]. The findings of this synthesis align with research on general critically ill patients regarding the need for consistent, respectful, and understandable information delivery [40, 42]. However, in neurocritical care settings, where considerable prognostic uncertainty prevails, more skillful information delivery strategies are required [5, 41]. Balancing the need for hope as a coping mechanism with avoiding unrealistic expectations is a delicate and challenging task [43]. To address the “hope-information paradox,” various communication strategies used in oncology have been proposed as potential solutions. These include methods such as the “ask-tell-ask” approach, in which HSCPs ask patients about their understanding, provide information, and then ask again to ensure comprehension [44]. Additionally, strategies such as the “hope for the best, plan for the worst” approach aim to balance optimism with realistic planning. “I wish” statements are used to express empathy and acknowledge the patient’s emotional experience during difficult conversations [45, 46]. However, their applicability to stroke remains unclear because of different disease trajectories, necessitating further research.

In SDM, incorporating patient preferences is essential. This study discovered that SDM participants validate the preferences of patients with diminished decision-making capacity through various methods, including advance directives (ADs), reconstructing the patient’s wishes, and observing their responses to stimuli, with ADs being prioritized. Some surrogates find reassurance when ADs are clearly documented, viewing them as authoritative guidance during overwhelming times [15, 33]. However, ADs are often unavailable, and when they are available, they may not align with the patient’s current clinical situation [18, 29, 32, 38]. This is consistent with the findings of quantitative studies conducted in neurocritical care settings where ADs accessibility was low and had little discernible influence on the choice of treatment regimen, especially in formal documentation [47–49]. To address these challenges, stroke-specific ADs have been developed [50]. However, as Morrison [51] noted, health care decisions are not simple, logical, or linear; they are complex, uncertain, emotionally charged, and subject to rapid change as the clinical situation evolves. Therefore, the emphasis should be on discussions on the conditions under which life is deemed worth living and collaborative assessment and deliberation among stakeholders at the moment of decision-making [48, 49].

This study described how key stakeholders perceive each other’s roles and their interactions in the SDM process, highlighting the multiple roles of family members. Although ADs, substituted judgment, and patient’s best interests are theoretically or legally valid standards for surrogate decision-making [52], family surrogates are inevitably influenced by their own cognitive and emotional factors, sometimes incorporating self-interest into their decisions [53, 54]. This can lead to irrational decisions not in the patient’s best interests, adding challenges for HSCPs. Additionally, family surrogates may face family vicissitudes and require care themselves [32, 55]. Thus, driven by clinical morality, HSCPs strive to provide support while balancing participation and responsibility to alleviate the emotional burden of decision-making on families [30, 32]. Researchers emphasized the importance of careful trade-offs when addressing the multiple roles of family members in SDM [15, 30, 32, 33, 47]; however, there is limited research on role dynamics among stakeholders in neurocritical care SDM, prompting further exploration.

The included studies revealed the significant role of religious and cultural factors in decision-making [15, 18, 29, 36]. On the positive side, patients and families often draw strength and hope from their religious faith. However, these beliefs can also lead to differences of opinion among those involved in SDM [15, 18]. In a review of LST in patients with disorders of consciousness, the authors highlight that religious beliefs provide both support and a source of conflict [56]. Regional and racial variations in LST practices and palliative care have also been observed in previous studies [57, 58]. Understanding the impact of sociocultural contexts is crucial to promote mutual understanding and prevent conflicts in SDM. It is worth noting that all 18 studies in this review are from Europe and the United States, indicating cultural homogeneity in the current research landscape. Compared to Western countries, the typical cultural characteristics of Confucianism, such as “familism” and “filial piety,” may significantly influence stakeholders’ behavior, resulting in distinct SDM patterns [59]. In addition, economic conditions, accessibility of health resources, and legislative and regulatory factors can significantly impact the decision-making process [60, 61]. Therefore, there is an urgent need for further research in socioculturally diverse settings to enrich the evidence on SDM in neurocritical patients with stroke.

This review has several limitations. Firstly, this study synthesized primary qualitative research through a rigorous process, but it does acknowledge there is a level of interpretation within both the primary and the synthesized findings. Secondly, the process was not conducted entirely in parallel by research team members, despite efforts to minimize potential bias through pilot methods and multiple rounds of team checks and discussions. Thirdly, language limitations within the research team constrained the literature search to English and Chinese, potentially overlooking valuable relevant literature published in other languages. Lastly, as discussed earlier, all included articles were from Europe and the United States, which may limit their applicability to sociocultural contexts outside these regions.

## Conclusions

For neurocritical patients with stroke, the SDM process is a complex balancing act heavily influenced by prognostic uncertainty. This process involves managing intricate role dynamics and facilitating information exchange among a tripartite decision-making body, all while being shaped by a broader sociocultural environment. Further research on stroke-specific communication strategies is urgently needed, particularly regarding the delivery of prognostic information. The complex role dynamics among SDM stakeholders, especially the multiple roles of family members, demand careful attention. The conceptual model developed from this review offers a valuable theoretical framework for researchers to further explore and understand SDM in neurocritical care settings. We recommend using this model as a foundation for additional empirical studies to build a more robust evidence base, particularly in diverse sociocultural contexts.

## Appendix

Keywords used in each string.

**Table:**

Population	AND	Phenomena of Interest	AND	Context
Stroke*		Shared Decision Making		Neurocritical Care
OR		OR		OR
Cerebral Infarction*		Decision mak*		Intensive care
OR		OR		OR
Brain Infarction*		Decision Support*		Critical care
OR		OR		OR
Cerebral Hemorrhage*		Decision Aid*		Acute phase*
OR		OR		OR
Cerebral Haemorrhage*		Preference*		Acute Care*
OR		OR		OR
Intracerebral Hemorrhage*		Choice*		Acute stroke care*
OR		OR		OR
Intracerebral Haemorrhage*		Participation		ICU
OR		OR		OR
Brain Hemorrhage*		Surrogate*		HDU
OR		OR		OR
Brain Haemorrhage*		goal of care*		Higher Dependen*
OR		OR		OR
Subarachnoid Hemorrhage*		goals of care*		Palliative care
OR		OR		OR
Subarachnoid Haemorrhage*		goal-setting		Terminal care
OR		OR		OR
Cerebrovascular Accident*		uncertainty		Life support Care
OR				
Cerebrovascular Disorder*				
OR				
Acute brain injur*				

Search record.

PubMed: 22.08.23.

**Table:**

#	Query	Results
#1	"Stroke"[Mesh] OR "Cerebral Infarction"[Mesh] OR "Cerebral Hemorrhage"[Mesh] OR "Subarachnoid Hemorrhage"[Mesh] OR Stroke* OR “Cerebral Infarction*” OR “Brain Infarction*” OR “Cerebral Hemorrhage*” OR “Cerebral Haemorrhage*” OR “Intracerebral Hemorrhage*” OR “Intracerebral Haemorrhage*” OR “Intracranial Hemorrhage*” OR “Intracranial Haemorrhage*” OR “Brain Hemorrhage*” OR “Brain Haemorrhage*” OR “Subarachnoid Hemorrhage” OR “Subarachnoid Haemorrhage” OR “Cerebrovascular Accident*” OR “Cerebrovascular Disorder*”OR “acute brain injur*”	538,724
#2	"Decision Making"[Mesh] OR "Decision Support Techniques"[Mesh] OR "Patient Participation"[Mesh] OR "Patient Preference"[Mesh] OR “shared decision making” OR “decision mak*” OR “Decision Support*” OR “Decision Aid*” OR “Surrogate*” OR “preference*” OR Choice* OR “participation” OR “goal of care” OR “goals of care*” OR goal-setting OR uncertainty	1,387,629
#3	"Critical Care"[Mesh] OR "Intensive Care Units"[Mesh] OR ("Life Support Care"[Mesh:NoExp]) OR "Terminal Care"[Mesh] OR "Palliative Care"[Mesh] OR “Neurocritical Care*” OR “Critical care*” OR “intensive care*” OR “Acute phase*” OR “Acute Care*” OR “acute stroke care” OR ICU OR HDU OR “Higher Dependen*” OR “Palliative care*” OR “life support care*” OR “terminal care*”	783,512
#4	#1 AND #2 AND #3	2,190
#5	Filters: Chinese, English	2,080

CINAHL: 22.08.23.

**Table:**

#	Query	Results
S1	(MH "Stroke") OR (MH "Intracranial Hemorrhage") OR Stroke* OR “Cerebral Infarction*” OR “Brain Infarction*” OR “Cerebral Hemorrhage*” OR “Cerebral Haemorrhage*” OR “Intracerebral Hemorrhage*” OR “Intracerebral Haemorrhage*” OR “Intracranial Hemorrhage*” OR “Intracranial Haemorrhage*” OR “Brain Hemorrhage*” OR “Brain Haemorrhage*” OR “Subarachnoid Hemorrhage” OR “Subarachnoid Haemorrhage” OR “Cerebrovascular Accident*” OR “Cerebrovascular Disorder*” OR “acute brain injur*”	164,626
S2	(MH "Decision Making") OR (MH "Goal-Setting") OR (MH "Patient Preference") OR “shared decision making” OR “decision mak*” OR “Decision Support*” OR “Decision Aid*” OR “Surrogate*” OR “preference*” OR Choice* OR “participation” OR “goal of care” OR “goals of care*” OR goal-setting OR uncertainty	463,024
S3	(MH "Critical Care") OR (MH "Acute Care") OR (MH "Life Support Care") OR (MH "Terminal Care") OR (MH "Palliative Care”) OR “Neurocritical Care*” OR “Critical care*” OR “intensive care*” OR “Acute phase*” OR “Acute Care*” OR “acute stroke care” OR ICU OR HDU OR “Higher Dependen*” OR “Palliative care*” OR “life support care*” OR “terminal care*”	253,971
S4	S1 AND S2 AND S3	648
S5	Narrow by Language: English and Chinese	632

EMBASE: 22.08.23.

**Table:**

#	Query	Result
#1	'cerebrovascular accident'/exp OR 'brain infarction'/exp OR 'brain hemorrhage'/exp OR 'subarachnoid hemorrhage'/exp OR Stroke* OR “Cerebral Infarction*” OR “Brain Infarction*” OR “Cerebral Hemorrhage*” OR “Cerebral Haemorrhage*” OR “Intracerebral Hemorrhage*” OR “Intracerebral Haemorrhage*” OR “Intracranial Hemorrhage*” OR “Intracranial Haemorrhage*” OR “Brain Hemorrhage*” OR “Brain Haemorrhage*” OR “Subarachnoid Hemorrhage” OR “Subarachnoid Haemorrhage” OR “Cerebrovascular Accident*” OR “Cerebrovascular Disorder*” OR “acute brain injur*”	854,618
#2	'shared decision making'/exp OR 'decision support system'/exp OR 'patient participation'/exp OR 'patient preference'/exp OR “shared decision making” OR “decision mak*” OR “Decision Support*” OR “Decision Aid*” OR “Surrogate*” OR “preference*” OR Choice* OR “participation” OR “goal of care” OR “goals of care*” OR goal-setting OR uncertainty	1,780,557
#3	'intensive care unit'/exp OR 'intensive care'/exp OR 'terminal care'/exp OR “Neurocritical Care*” OR “Critical care*” OR “intensive care*” OR “Acute phase*” OR “Acute Care*” OR “acute stroke care” OR ICU OR HDU OR “Higher Dependen*” OR “Palliative care*” OR “life support care*” OR “terminal care*”	1,813,821
#4	#1 AND #2 AND #3	7,490
#5	[embase]/lim NOT ([embase]/lim AND [medline]/lim)	3,511
#6	([chinese]/lim OR [english]/lim)	3,403

PsycINFO: 22.08.23.

**Table:**

#	Query	Result
S1	DE "Cerebrovascular Accidents" OR DE "Cerebral Infarction" OR DE "Cerebral Hemorrhage" OR DE "Subarachnoid Hemorrhage" OR Stroke* OR “Cerebral Infarction*” OR “Brain Infarction*” OR “Cerebral Hemorrhage*” OR “Cerebral Haemorrhage*” OR “Intracerebral Hemorrhage*” OR “Intracerebral Haemorrhage*” OR “Intracranial Hemorrhage*” OR “Intracranial Haemorrhage*” OR “Brain Hemorrhage*” OR “Brain Haemorrhage*” OR “Subarachnoid Hemorrhage” OR “Subarachnoid Haemorrhage” OR “Cerebrovascular Accident*” OR “Cerebrovascular Disorder*”OR “acute brain injur*”	55,628
S2	DE "Decision Making" OR DE "Decision Support Systems" OR DE "Client Participation" OR DE "Uncertainty" OR “shared decision making” OR “decision mak*” OR “Decision Support*” OR “Decision Aid*” OR “Surrogate*” OR “preference*” OR Choice* OR “participation” OR “goal of care” OR “goals of care*” OR goal-setting OR uncertainty	569,050
S3	DE "Intensive Care" OR DE "Critical Period" OR DE "Palliative Care" OR “Neurocritical Care*” OR “Critical care*” OR “intensive care*” OR “Acute phase*” OR “Acute Care*” OR “acute stroke care” OR ICU OR HDU OR “Higher Dependen*” OR “Palliative care*” OR “life support care*” OR “terminal care*”	55,552
S4	S1 AND S2 AND S3	244
S5	Narrow by Language: English	236

Web of Science: 22.08.23.

**Table:**

#	Query	Results
#1	Stroke* OR “Cerebral Infarction*” OR “Brain Infarction*” OR “Cerebral Hemorrhage*” OR “Cerebral Haemorrhage*” OR “Intracerebral Hemorrhage*” OR “Intracerebral Haemorrhage*” OR “Intracranial Hemorrhage*” OR “Intracranial Haemorrhage*” OR “Brain Hemorrhage*” OR “Brain Haemorrhage*” OR “Subarachnoid Hemorrhage” OR “Subarachnoid Haemorrhage” OR “Cerebrovascular Accident*” OR “Cerebrovascular Disorder*” OR “acute brain injur*”	506,653
#2	“shared decision making” OR “decision mak*” OR “Decision Support*” OR “Decision Aid*” OR “Surrogate*” OR “preference*” OR Choice* OR “participation” OR “goal of care” OR “goals of care*” OR goal-setting OR uncertainty	2,826,611
#3	“Neurocritical Care*” OR “Critical care*” OR “intensive care*” OR “Acute phase*” OR “Acute Care*” OR “acute stroke care” OR ICU OR HDU OR “Higher Dependen*” OR “Palliative care*” OR “life support care*” OR “terminal care*”	384,347
#4	#1 AND #2 AND #3	1,199
#5	#1 AND #2 AND #3 and English (Languages)	1,141
